# Human Rights in Older Age: A Critical Reflection of the Debate Around a UN Convention on the Rights of Older Persons

**DOI:** 10.1093/geroni/igaa057.063

**Published:** 2020-12-16

**Authors:** Stefan Hopf, Federica Previtali, Nena Georgantzi

**Affiliations:** 1 National University of Ireland Galway, Galway, Ireland; 2 Tampere University, Tampere University, Finland; 3 Age Platform Europe, Brussels, Brussels Hoofdstedelijk Gewest, Belgium

## Abstract

In recent years ageism has received increase international attention. In 2016 the UN dedicated the International Day of Older Persons to the fight against ageism and the World Heath Organization launched a campaign to combat ageism. This growing interest is also illustrated by the establishment of the UN Open-Ended Working Group on Ageing, and the related work on a UN convention on the rights of older persons, which, among other things, aims to provide better protection against discrimination. The ongoing discussions about a convention is accompanied by tensions between views assuming an older persons’ specific convention may reproduce age-related group differences and could perpetuate ageism, and those who argue that it will help reducing it. This article critically reflects on these discussions and some aspects of a potential convention that could provide basis for ageism critique. We refer to central sociological and legal arguments of the debate around ageism and age-based distinctions, which show clear intersections, e.g. the legal discussion one the justifiability of the general use of "age limits" and the socio-scientific debate on the relationship between age categorization and ageism. These intersections serve as central starting point for the question whether and to what extent age group differentiation and targeted human rights protection may (re-)produce ageism. Finally, we argue in favor of re-framing the debate about a convention on the rights of older persons towards a more universalist approach, which addresses possible age inequalities and critically reflects on the connection between chronological age and targeted human rights provisions.

